# An insidious rectus abdominis muscle injury in an elite volleyball player: a case report

**DOI:** 10.1186/s13256-023-04299-w

**Published:** 2024-01-23

**Authors:** Federico Giarda, Diego Contro, Maurizio Fasano, Mirko Poli, Maurizio Giacchino

**Affiliations:** 1 Rehabilitation Medicine and Neurorehabilitation Unit, Neuroscience Department, A.S.S.T. Grande Ospedale Metropolitano Niguarda, Piazza Dell’Ospedale Maggiore 3, Milan, Italy; 2 Technical Director at Medical Lab, Sport Medicine and Physiotherapy Center, Piazza Luigi Rey, Turin, Italy; 3 Sport Medicine and Physiotherapy Center, Medical Lab Asti, 226 Alessandria, Asti Italy; 4 Orthopedics and Traumatology Unit, Emergency and Urgency Department, A.S.S.T. Grande Niguarda, Piazza Maggiore 3, Milan, Italy

**Keywords:** Volleyball injuries, Muscular tear, Abdominal muscle, Ultrasound

## Abstract

**Background:**

Structural muscle injuries are characterized by acute and localized onset of pain. Abdominal muscle injuries are an insidious pathology in overhead athletes. However, only a few cases are reported in literature related to volleyball players, where clinical presentation may not have reflected the severity of the lesion.

**Case presentation:**

An elite volleyball player, a 21-year-old Caucasian female, reported the onset of mild abdominal muscular pain, confirmed on clinical evaluation findings and self-reported symptoms. Abdominal muscle ultrasound was performed following 2 weeks of continuing symptoms. This evidenced a more serious structural muscle injury of the rectus abdominis (type 3b). Having this correct diagnosis allowed a personalized rehabilitation program to be instituted to enable a safe return to play.

**Conclusion:**

In presence of persistent abdominal muscle pain, even if mild, the possibility of a structural muscle injury must be considered. Clinical evaluation must be complemented by an instrumental evaluation including an ultrasound by an experienced operator for correct diagnosis and the setting of functional recovery related to biological healing.

## Background

In volleyball the risk of injury is mainly related to ankle sprains and pathologies affecting the knee and shoulder, including jumping and the overhead motion of hitting the ball repetitively. However, muscle injuries in this population are rarely described in literature despite their clinical relevance [1]. Muscle injuries typically occur in biarticular muscles with a high percentage of type II fast-twitch fibers, especially during eccentric muscle contraction. However, in sports that involve overhead movements such as serve, smash, or spike as in volleyball or tennis, muscular tears can occur in the rectus abdominis, which is a monoarticular and multilaminate muscle [2]. The typical injury mechanism described in overhead athletes is an eccentric overload, followed by the forced contraction of the nondominant rectus abdominis during the cocking phase of the overhead service motion [3]. Acute indirect muscular injuries are classified in functional muscle disorders when an acute indirect muscle disorder is not associated with macroscopic evidence, from ultrasound (US) or magnetic resonance imaging (MRI), of muscular tear and structural muscle injuries. Structural muscle injuries are typically associated with sudden-onset and functional muscle strength loss and localized pain, aggravated by both pressure and contrasted contraction with a stretch-induced pain aggravation. Notably, partial muscle tears that are minor or moderate are classified as type 3A and 3B, respectively, with minor partial tears having a maximum diameter less than and moderate partial tears having a diameter greater than a muscle fascicle/bundle [4].

## Case presentation

**Fig. 1:**
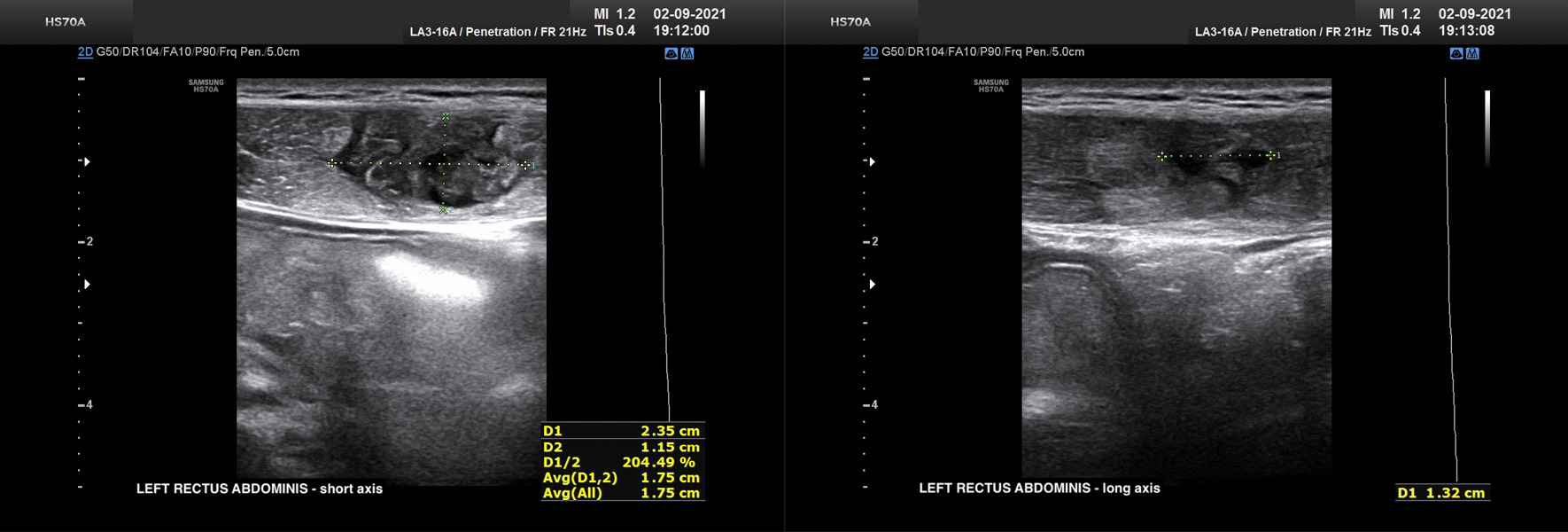
At the level of the distal myotendinous junction of the first muscle portion of the left rectus abdominis, a discontinuity can be seen in an irregularly hypo-anechoic area with fragments of isoechoic tissue inside it. The area measures approximately 19 × 23.5 × 11.5 mm (longitudinal × transverse × anteroposterior diameters, respectively), shows patterns of moderate hypervascularization on power Doppler, and demonstrates diastasis of the margins during the execution of functional tests of 1/2 crunch and especially with legs raised. The lesion affects the anteroposterior diameter for approximately 85% of the entire thickness. The finding depicts a II-degree lesion (3B according to the Munich consensus 2012) of the left rectus abdominis with residual intramuscular hematoma in a predominantly fluid phase

In 2021, a 21-year-old elite Caucasian female volleyball player, playing for her national team as a spiker, where her role involved multiple tasks, including spiking, blocking, and defense, presented with tenderness to the left proximal part of the rectus abdominis, on 10 August. The patient reported no previous history of muscle injury, and no physical restrictions on symptom presentation. However, pain onset was experienced more acutely during sporting activity, when playing in international competitions. Due to the low intensity of the pain, with no limitations in sporting activity, the athlete was allowed to play by reducing the number of attacks on court in each session of training. Some weeks following this, due to persistence of symptoms, especially present during prolonged physical activity, on 27 August, an ultrasound evaluation was performed. A structural tear was detected at the level of the distal myotendinous junction of the proximal muscle belly of the left rectus abdominis. The player was benched, and subsequently, the player was sent to us for follow-up and for rehabilitation treatment in preparation for the start of the regular volleyball season. On clinical reevaluation she presented modest pain on palpitation of the proximal left rectus abdominis muscle, and concentric contraction and stretching caused discomfort, but not pain to the area. The patient reported no pain being experienced during daily activities, including walking and running. However, a reassessment by ultrasound on 2 September evidenced a left rectus abdominis injury with intramuscular hematoma, consistent with a partial muscle tear, type 3b according to the Munich consensus [4]. The site of pain indicated by the patient showed an area of loss of muscle echo pattern, approximately 24 × 11 × 13 mm, filled with fluid. No hematoma was detectable external to the muscle belly or within the rectus sheath (Fig. 1).

**Fig. 2:**
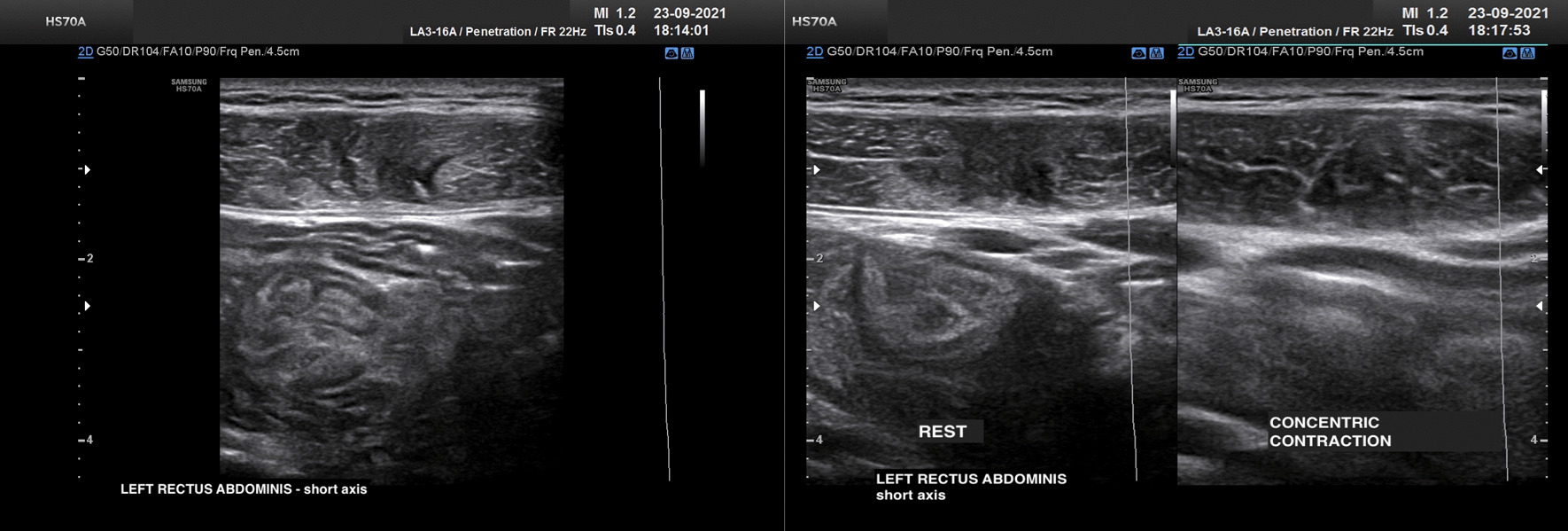
The ultrasound shows the apposition of tissue with varying degrees of echogenicity (from hypoechoic to iso-hyperechoic), which completely rehabilitates the area of discontinuity. Power Doppler hypervascularization patterns are not observed. The execution of the functional tests of concentric activation of the rectus abdominis (crunch and leg raise) highlights regular tensioning of the repair tissue with residual, minimal response to reduced resistance in the context of the hypoechoic tissue alone. The finding depicts the results of II-degree stabilized lesion (3B according to the Munich consensus 2012) of the left rectus abdominis in the stabilization phase

Following the ultrasound, the recovery program was initiated. In the first week, the recovery program included physical and manual therapies aimed at controlling pain. Simultaneously, with the aim of preventing deconditioning, the athlete continued strengthening muscles and associated movements functionally unrelated to the area of injury, including the exercise bike for aerobic training. From the second week, active exercises were intensified, including postural control exercises with indirect activation of the abdominal wall muscles. From the third week, direct abdominal muscle strengthening exercises were introduced, avoiding movements that could cause any type of pain at the injury site. In addition, specific sports exercises for the defense and reception game were started, avoiding activities placing excessive strain on the abdominal muscles such as blocking and jumping. An ultrasound examination was performed before moving on to the last stage of rehabilitation on 23 September. It evidenced the full reabsorption of the hematoma noted in the first evaluation, and the advanced repair of the muscle injury on both static and dynamic evaluation (Fig. 2).

**Fig. 3:**
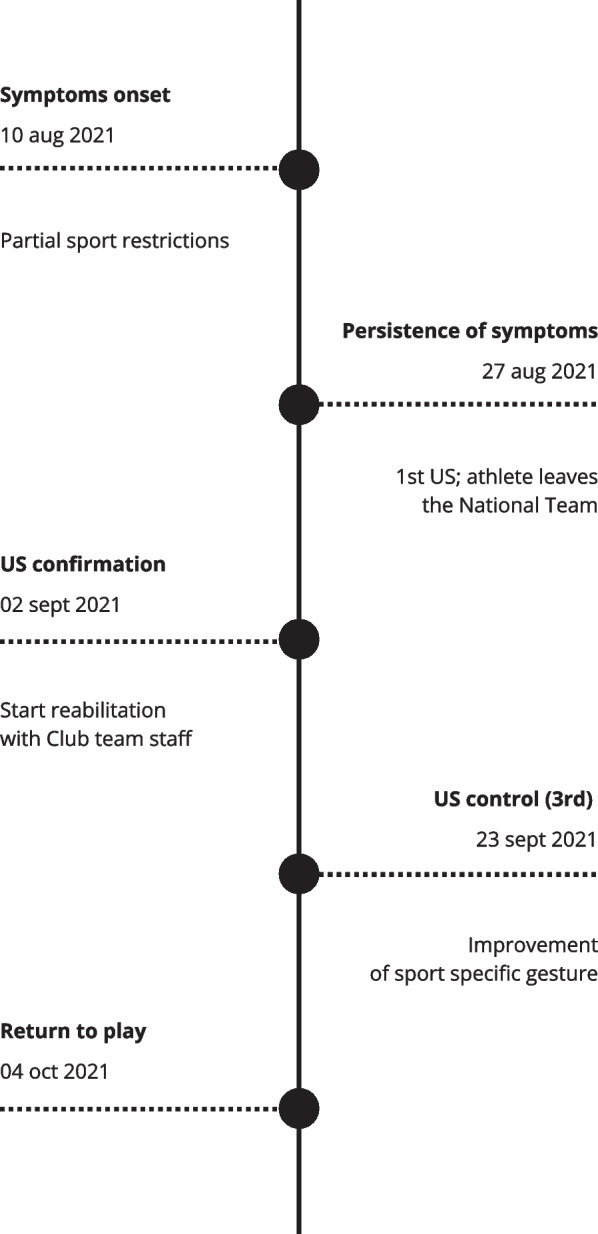
Timeline representing the chronology of events

In the fourth week, all the role-specific movements were gradually reintroduced until complete return to play without limitations was achieved (Fig. 3). Diet and supplementation were also monitored during the rehabilitation period, this, to optimize recovery and maintain body composition. The athlete was prescribed a well-balanced diet that included adequate amounts of protein that play a key role in this phase of rehabilitation, carbohydrates, antioxidants, and moderate fats. Daily supplements by month were prescribed for decreasing inflammation and ameliorating the anabolic signals, including omega 3, 4 g a day, creatine monohydrate, 5 g a day, and essential amino acids (EAA), 5 g twice a day. (5, 6).

The patient was amazed at the initial ultrasound findings being so serious, as they were unexpected, considering the minor symptoms she experienced. However, the patient demonstrated full compliance to the proposed treatment plan, respecting biological and rehabilitation times, for a safe return to play.

During peak season the patient maintained an exercise program to prevent reinjury, continuing sporting activity without interruption until the end of the season in April 2022.

## Discussion and conclusion

There is little published research in literature to date describing muscular injuries in volleyball players. Other studies have previously highlighted how injuries affecting the abdominal wall are reported as common, while epidemiological studies, especially those related to volleyball, do not support this [7]. Beer *et al.* analyzing data from the International Volleyball Federation (FIVB) Injury Surveillance System (ISS) reported 6/440 abdominal wall injuries, over a 48-month observational period (4.1%) [8]. Despite not being frequent in our clinical practice, the number of muscle injury cases resulting from playing volleyball is not negligible; however, epidemiological articles on volleyball-related injuries rarely include muscular and abdominal injuries [9]. One article was found that discussed injuries sustained by professional beach volleyball players and reported the injury as requiring medical attention even though no withdrawal from sports activities was required [10]. Previous articles have been published on rectus abdominis injury in overhead athletes, including tennis and handball players, although lesions were observed in most cases at the contralateral rectus abdominis of the armed wing, generally at the infraumbilical level [11, 12]. As described above, the patient’s lesion was on the opposite side with respect to the dominant limb, although at a proximal level. Notably, this case concerns the paucity of the associated symptomatology and the absence of a specific onset of pain. The patient instead reported a substantial stability of mild symptoms, which did not appear typical of significant muscle damage. These features are atypical of a grade 3b structural injury, although the characteristics of the abdominal wall musculature differ from the biarticular muscles, being the expected site of muscle lesions in other sports, as previously described. The onset of symptoms, in addition to being atypical, occurred during an international competition, where the staff did not have the usual support for diagnostics. This, together with the minor symptoms presented, may have influenced the initial delay in diagnostic imaging. Furthermore, when the evaluation was done due to suspected injury, the symptoms and clinical signs did not indicate a medium–high-grade tear; thus, an ultrasound was performed by an experienced operator to confirm a diagnosis. Ultrasonography is the instrument of choice due to its accessibility and the possibility to evaluate the dynamic response of the muscle. In addition, closely monitoring the hematoma following a muscle injury is essential to preemptively identify any dystrophic calcifications that over time can become mature bone (heterotopic ossification) causing pain or functional limitation [13]. The same ultrasonographer monitored progress and healing at follow-up to avoid ultrasound examination variability in the results.

Persistent localized abdominal muscle wall pain, even of low intensity, should always be investigated using instrumental examination. The use of ultrasonography by an operator experienced in evaluating elite athletes is considered paramount in making an accurate diagnosis, and to continuously evaluate both static and dynamic conditions as well as muscle injury evolution.

A global and multidisciplinary approach permitted a complete recovery and return to play without complications approximately 30 days after the first evaluation. This is in accordance with the recovery times for this grade of injury as reported in the literature.

## Data Availability

All relevant data are within the manuscript.

## Abbreviations

EAA: Essential amino acids
FIVB: International Volleyball Federation
ISS: Injury Surveillance System
